# Design and Implementation of a Sun Outage Simulation System with High Uniformity and Stray Light Suppression Capability

**DOI:** 10.3390/s25154655

**Published:** 2025-07-27

**Authors:** Zhen Mao, Zhaohui Li, Yong Liu, Limin Gao, Jianke Zhao

**Affiliations:** 1 Xi’an Institute of Optics and Precision Mechanics, Chinese Academy of Sciences, Xi’an 710119, China; maozhen2017@opt.ac.cn (Z.M.); liuyong@opt.ac.cn (Y.L.); glm@opt.ac.cn (L.G.); zjk@opt.ac.cn (J.Z.); 2 University of Chinese Academy of Sciences, Beijing 100049, China

**Keywords:** Sun outage, stray light, solar radiation, optical trap, spectrum match, irradiance uniformity

## Abstract

**Highlights:**

**What are the main findings?**

- A solar radiation simulation system with adjustable wide dynamic range (2.5–130 mW), high irradiance uniformity (85.8%), and spectral matching (78%) was developed for simulating Sun outage conditions in the 1540–1560 nm communication band.
- A combined homogenization structure using multimode fiber and apodizer was proposed to achieve flat-top large-aperture beam shaping in the far field.
- A power–spectrum coordinated optimization strategy was proposed for filter design, enabling stable spectral reconstruction under varying power levels.
- Through mirror surface roughness control and the implementation of a U-shaped light trap, the total stray light level was suppressed to the 10^−12^ W scale.

**What are the implications of the main findings?**

- The system provides a standardized and quantifiable test platform for evaluating satellite laser communication terminals under simulated Sun outage conditions.
- It fills a technological gap in ground-based validation of solar interference effects on laser communication, supporting future spaceborne link verification and design optimization.

**Abstract:**

To enable accurate evaluation of satellite laser communication terminals under solar outage interference, this paper presents the design and implementation of a solar radiation simulation system targeting the 1540–1560 nm communication band. The system reconstructs co-propagating interference conditions through standardized and continuously tunable output, based on high irradiance and spectral uniformity. A compound beam homogenization structure—combining a multimode fiber and an apodizator—achieves 85.8% far-field uniformity over a 200 mm aperture. A power–spectrum co-optimization strategy is introduced for filter design, achieving a spectral matching degree of 78%. The system supports a tunable output from 2.5 to 130 mW with a 50× dynamic range and maintains power control accuracy within ±0.9%. To suppress internal background interference, a *BRDF*-based optical scattering model is established to trace primary and secondary stray light paths. Simulation results show that by maintaining the surface roughness of key mirrors below 2 nm and incorporating a U-shaped reflective light trap, stray light levels can be reduced to 5.13 × 10^−12^ W, ensuring stable detection of a 10^−10^ W signal at a 10:1 signal-to-background ratio. Experimental validation confirms that the system can faithfully reproduce solar outage conditions within a ±3° field of view, achieving consistent performance in spectrum shaping, irradiance uniformity, and background suppression. The proposed platform provides a standardized and practical testbed for ground-based anti-interference assessment of optical communication terminals.

## 1. Introduction

In recent years, laser communication has emerged as a key enabler of space-based high-speed, high-bandwidth, and low-latency communication. Compared with traditional RF systems, laser links offer superior spectral efficiency and robustness, particularly in deep-space exploration and satellite networking applications [1,2,3,4]. However, during Sun outage events—when the Sun, satellite, and ground terminal become nearly collinear—the intense solar background radiation couples into the optical link, drastically reducing the signal-to-noise ratio and potentially interrupting communication [5,6].

To address Sun outages, various mitigation strategies have been explored globally. For instance, Vineet K. Srivastava et al. (2022) developed a geometrical Sun outage prediction model for Earth-orbiting satellites, enabling easier prediction of spacecraft solar outage over ground station antennas using state vector or TLE information [7]. Li et al. (2024) introduced an all-optical approach utilizing an optical parametric amplification (OPA) processor to suppress the impact of Sun outages in laser satellite communication systems [8]. Their optimized OPA processor demonstrated a significant improvement in signal quality during solar interference events. Additionally, Jin et al. (2024) proposed a Local Pre-Rerouting Algorithm (LPRA) for inter-satellite links in low Earth-orbit satellite networks, aiming to reroute services before Sun outages occur, thereby enhancing network resilience [9]. Furthermore, some countries have conducted in-orbit experiments to investigate Sun outage effects [10,11,12], including China’s Tianwen-1 mission, ESA’s Laser Communication Terminal (LCT) project, and NASA’s Laser Communications Relay Demonstration (LCRD). These studies indicate that solar noise has minimal impact on communication when the Sun–Earth–Probe (SEP) angle exceeds 3°. From a methodological perspective, existing anti-interference approaches can be categorized as follows: Array detector systems [13] employ spatial diversity to enhance signal-to-noise ratio, while adaptive phase correction [14] and simplified modulation schemes [15] optimize signal recovery in hostile channels. While the former dominates in-orbit applications, the latter is critical for ground validation systems where realistic solar interference must be replicated without distorting the co-propagating signal.

Although prior research has confirmed general Sun outage effects under wide-angle conditions, there remains a lack of high-precision and repeatable ground-based verification platforms for evaluating the anti-interference performance of terminals within narrow fields of view, such as ±3°. Most existing studies rely on costly in-orbit resources with limited controllability and poor repeatability, making them unsuitable for early-stage engineering validation. Moreover, conventional solar simulators are mostly static simulators that only simulate the spectral energy distribution of solar radiation [16,17,18,19]. The solar simulator discussed in this paper is designed for solar transit scenarios, which is a dynamic simulator integrating relative attitude changes, a certain field of view range, spectral characteristics, and specific irradiance energy.

To address these gaps, this paper presents the design and implementation of a high-consistency solar radiation simulation system targeting the 1540–1560 nm communication band. The system features high spectral fidelity, superior irradiance uniformity, and wide-range power adjustability, and for the first time integrates signal beam co-path output and stray light suppression mechanisms to reconstruct realistic Sun outage interference scenarios on the ground. The platform supports quantitative anti-jamming evaluation of optical terminals over a ±3° field of view. The main innovations are the following: (1) Far-field high-uniformity beam shaping: A composite beam homogenization scheme using a multimode fiber and apodizer achieves far-field 85.8% irradiance uniformity over a 200 mm aperture; (2) Spectral power co-optimized filter design: A joint power-spectrum optimization model improves spectral consistency across the communication band, achieving a match rate of 78%; (3) Stray light modeling and suppression: A *BRDF*-based simulation framework identifies dominant scattering paths, sets a 2 nm roughness threshold for key optics, and uses a U-shaped light trap to reduce background stray light to 5.13 × 10^−12^ W.

## 2. Overall System Design

### 2.1. Functional Requirements

In satellite laser communication, the Sun-transit phenomenon refers to the alignment of the Sun, satellite, and ground terminal, during which strong solar background radiation enters the receiver’s field of view, resulting in a sharp drop in the signal-to-noise ratio (SNR) and possible communication interruption. This phenomenon is periodic and transient, with interference typically lasting several minutes. Sunlight in the spectral neighborhood of the 1550 nm communication band (1540–1560 nm) still emits considerable radiance, posing significant interference risks—particularly under low signal power conditions or with large receiving apertures.

To investigate system-level responses under such conditions, a ground-based simulation platform is required to generate solar background radiation with high spectral consistency, tunable intensity, and spatial uniformity. Coupled with a weak signal generation module, such a system should recreate co-propagated interference scenarios within narrow angular fields. The design must fulfill the following key requirements: (1) spectral output matching the communication band while accounting for source wavelength and power variation; (2) continuously adjustable irradiance to simulate various interference levels; (3) uniform far-field beam profile across the receiver field of view; and (4) support for ±3° incident angle modulation to mimic dynamic solar alignment during transit events.

### 2.2. Performance Requirements

To meet the above-mentioned simulation requirements, the system developed in this study aims to generate high-consistency, tunable, and reconfigurable solar background radiation within the communication band, while supporting spatial co-propagation with weak signal beams. The design focuses on achieving three key performance objectives: (1) Spectral consistency: A multilayer optical filter is optimized to compensate for the nonlinear power–wavelength behavior of the laser source, maintaining stable spectral output within the communication window, with a target spectral matching degree of no less than 75%. (2) Far-field irradiance uniformity: A hybrid beam shaping structure, combining a homogenizing fiber and a beam truncation element, is employed to produce a flat-top beam with over far-field 85% irradiance uniformity across a 200 mm aperture. (3) Continuously tunable output power: A programmable attenuation system enables smooth power modulation from 10% to 100% (max = 130 mW), covering the full range of solar irradiance levels observed before, during, and after Sun-transit events. (4) Stray light suppression capability: Due to the co-propagation of solar background and communication signals within a shared optical path, multiple internal reflection and scattering events—especially from coated surfaces—can introduce stray light that degrades signal recognition. Therefore, the system is required to incorporate a theoretical model for stray light path analysis and implement spatial extinction structures in the design to limit background energy. The target performance is to maintain a signal-to-background ratio (SNR) greater than 10:1 under a 10^−10^ W signal output condition.

Additionally, a precision rotation stage allows for ±3° incident angle simulation, enabling reconstruction of dynamic alignment scenarios. The entire system is designed to support the physical simulation of co-aligned solar interference environments, with a focus on high spectral and spatial fidelity, wide dynamic output range, and low-background signal recognition capabilities.

### 2.3. System Architecture and Functional Module Division

The solar radiation simulation module is designed to reconstruct the spectral, spatial, and irradiance characteristics of solar interference during Sun-transit events. It integrates a tunable laser source, spectral shaping filter assembly, beam homogenization optics, collimation system, and a programmable attenuation unit. After emission, the laser beam is processed through a homogenizing fiber and a beam-flattening structure to generate a uniform far-field profile. A software-controlled attenuation module enables continuous power adjustment from 10% to 100% (max = 130 mW), covering the irradiance levels corresponding to different Sun-transit phases. Additionally, a precision rotation stage is included to simulate incident angle variations within ±3°, representing the dynamic angular relationship between the Sun and the communication terminal.

The signal beam generation module provides ultra-weak narrowband laser output at 1550 nm, emulating satellite terminal transmission under strong solar background conditions. This module consists of a narrow-linewidth 1550 nm laser source, and a motorized translation stage. By adjusting the optical alignment and beam collimation via precise displacement control, the system simulates variable beam divergence angles. Combined with a fiber attenuator, this enables signal power adjustment at the 10^−10^ W level, supporting evaluations of signal detectability under varying background intensities.

**Figure 1:**
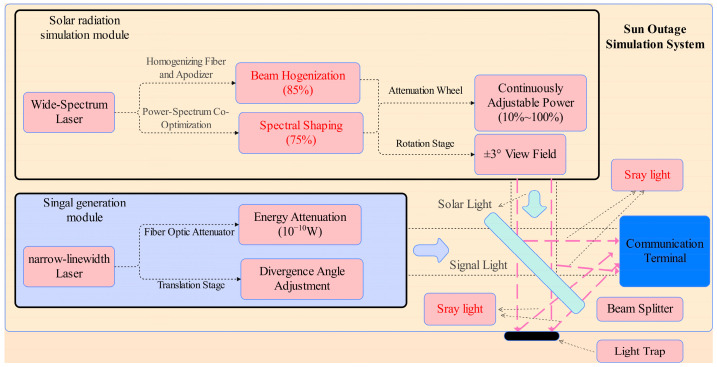
Schematic diagram of the system.( The red text annotations indicate the key technologies indicators and performance requirements of the system).

The two optical beams are combined via a semi-reflective beam splitter to form a shared propagation path, enabling spatial co-alignment of the solar background and signal beams. This configuration facilitates controlled reconstruction of the optical interference environment encountered during Sun-transit events, as illustrated in Figure 1, and provides a unified physical input condition for subsequent anti-interference capability testing. 

## 3. Critical Technical Approach

### 3.1. Large-Aperture, Far-Field Uniformity Modeling and Design

Conventional wavelength-specific homogenization techniques—such as diffractive optics, lens arrays, and spatial light modulators (SLMs)—are fundamentally constrained by chromatic aberrations, limited scalability, and power-handling inefficiencies, making them unsuitable for applications requiring simultaneous large-aperture and far-field uniformity [20,21,22,23,24,25,26]. In contrast, our fiber–apodizer architecture overcomes these limitations by exploiting controlled multimodal propagation in a tailored optical fiber, synergized with precision apodization for optimized beam shaping. This composite design achieves exceptional far-field intensity uniformity propagation while maintaining wavelength selectivity—a critical capability for advanced laser systems demanding joint spectral and spatial control. The shaping principle is based on the Multimode Interference–Mode Interference (MIMI) mechanism. The homogenizing fiber supports multiple guided modes, and when the input is a Gaussian beam, each guided mode undergoes phase evolution along distinct propagation constants within the fiber. The output optical field distribution E(x,y) is given by Equation (1):

(1) $$E(x,y)=\sum_{m=1}^{N}c_{m}\psi_{m}(x,y)e^{i\beta_{m}L}$$

where $\psi_{m}$ denotes the modal eigenfunction, $\beta_{m}$ represents the propagation constant, and L is the fiber length. Due to phase differences between the supported modes, the output field forms a spatially varying multimode coherent superposition. After temporal or statistical averaging, the output intensity distribution can be approximated by Equation (2), which averaging effectively mitigates central bias and power fluctuations in the input beam. However, residual high-order modes can still induce localized modulation near the beam edge, resulting in a non-uniform energy distribution in the peripheral region.

(2) $$I\approx \frac{1}{A}\iint_{\mathrm{core}}I_{in}(x,y)dxdy$$

where $I_{in}(x,y)$ denotes optical intensity distribution, and *A* denotes cross-sectional area of the fiber core.

To further suppress high spatial frequency components, an apodizing diaphragm with a specific aperture diameter *D* is introduced at the output end of the homogenizing fiber. This diaphragm physically truncates the edge components of the beam, functioning as a spatial frequency filter. The relationship between the homogenization performance $U_{E}(D)$ in the diaphragm diameter D is given by Equation (3):

(3) $$U_{E}(D)=1-\frac{\int_{r>D/2}I(r)dr}{\int_{0}^{R}I(r)dr}$$

where I(r) denotes optical intensity distribution within radius *r*, and *r* denotes the radius within which the uniformity requirement is satisfied.

To verify the theoretical effectiveness of the beam homogenization scheme, the energy distribution at the equivalent focal plane of the homogenizing fiber was measured and analyzed. Theoretical calculations indicate that within the central 30% region of the output beam diameter, the irradiance uniformity can reach up to 90%. This result provides design constraints for determining the optimal diaphragm size.

**Figure 2:**
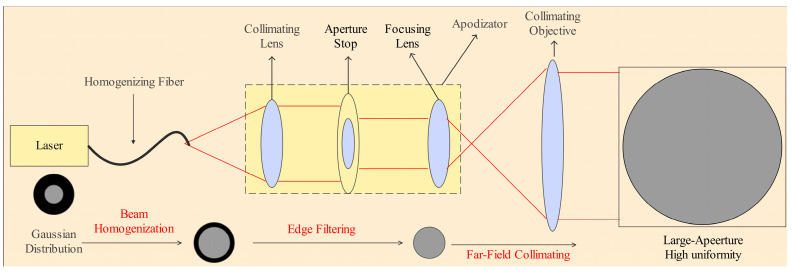
Schematic diagram of large-aperture far-field beam homogenization optics. (The red annotations highlight the critical steps in the proposed homogenization method.)

During the theoretical design phase, to ensure that the irradiance uniformity within the central region exceeds 90%, the optimal diameter range of the apodizing diaphragm D was determined to be between 15 mm and 40 mm. Within this range, high-frequency modal structures at the beam edges can be effectively filtered out, significantly improving spatial energy uniformity at the cost of a slight reduction in total transmitted energy. The specific dimensions of the apodizer used in the experimental setup are detailed in the following experimental section. The working principle of the homogenization structure combining the fiber and apodizer is illustrated in Figure 2.

### 3.2. Design Method for Optical Filters Addressing Power–Spectrum Nonlinearity Characteristics

**Figure 3:**
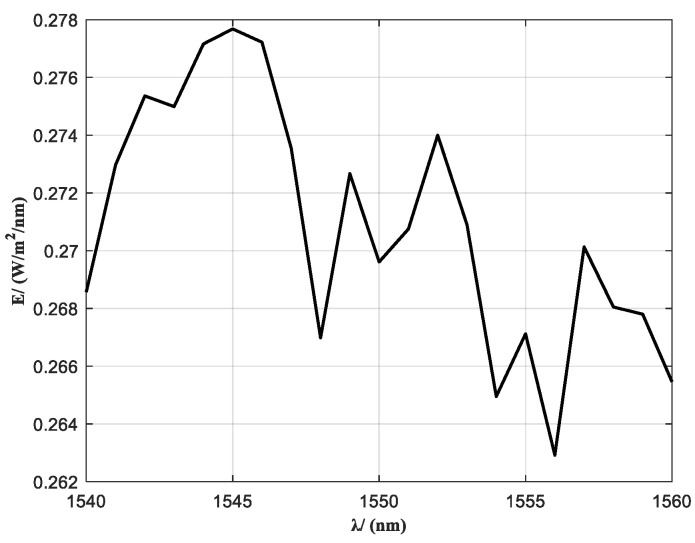
Standard solar spectrum.

In a Sun outage interference simulation system, the spectral characteristics of the solar radiation background are among the core parameters determining the realism of interference reconstruction. To achieve high-fidelity reproduction of the solar spectral distribution within the communication band (1540–1560 nm), as illustrated in Figure 3, the light source must spectrally match the actual solar spectral radiance to ensure effective spectral-domain coupling between the signal and interference beams. However, conventional spectral filter design is typically based on static source spectra, which makes it unsuitable for handling the nonlinear spectral response of laser sources under varying power levels [27,28].

To address this, a spectral filter design strategy based on the co-optimization of laser power–spectrum characteristics is proposed. Using the standard solar spectral radiance in the communication band as the target function, the filter’s wavelength-dependent transmittance T(λ) is optimized to modulate the original laser output $L_{\mathrm{source}}(\lambda ,P)$, producing the desired output spectrum $L_{\mathrm{source}}(\lambda ,P)\cdot T(\lambda )$. To achieve spectral shape consistency under normalization conditions, the filter’s transmittance must satisfy a spectral matching Equation (4):

(4) $$\frac{L_{\mathrm{source}}(\lambda ,P)\cdot T(\lambda )}{\mathrm{Max}[T(\lambda )\cdot L_{\mathrm{source}}(\lambda ,P)]}=\frac{L_{\mathrm{solar}}(\lambda )}{\mathrm{Max}[L_{\mathrm{source}}(\lambda )]}$$

a spectral uniformity index $U_{L}$ is introduced as the evaluation metric for filter performance, as defined in Equation (5):

(5) $$U_{L}=\frac{2*\mathrm{Min}(L_{j}^{i}\cdot T_{i})}{\mathrm{Min}(L_{j}^{i}\cdot T_{i})+\mathrm{Max}(L_{j}^{i}\cdot T_{i})}$$

where Min [] denotes the minimum operator, and Max [] denotes the maximum operator. The spectral response at each wavelength point $\lambda_{i}$ (i-th index) was measured under different power levels *P*_j_ (j-th index).

To achieve optimal matching performance, the filter design problem is formulated as a linear programming optimization, with the objective of minimizing the normalized error function. The following constraints are applied as Equation (6):

(6) $$\left\{\begin{array}{l} U_{L}\geq 80\%\\ {\mathrm{Min}}_{\lambda_{i}}\left[T(\lambda_{i})\right]\geq \varepsilon \\ 0\leq T(\lambda )\leq 1 \end{array}\right.(\varepsilon =0.2)$$

where the first constraint ensures that the spectral uniformity exceeds 80%; the second guarantees sufficient energy utilization efficiency; the third defines the physical bounds of the transmittance values. The actual transmittance design of the spectral filter is given by Equation (7):

(7) $$T_{i}(\lambda_{i})=\frac{L_{\mathrm{solar}}(\lambda_{i})}{L_{\mathrm{source}}(\lambda_{i},P_{j})]}\cdot \frac{\mathrm{Max}[L_{\mathrm{solar}}(\lambda_{i})]}{\mathrm{Max}[L_{\mathrm{source}}(\lambda_{i})]}$$

The final optimization objective is to minimize the normalized spectral error function, which is expressed as Equation (8):

(8) $$\mathrm{Min}(\sum_{j=1}^{m}\sum_{i=1}^{n}\left|\frac{L_{\mathrm{source}}(\lambda_{i},P_{j})\cdot T(\lambda_{i})}{\sum L_{\mathrm{source}}(\lambda_{i},P_{j})\cdot T(\lambda_{i})}-\frac{L_{\mathrm{solar}}(\lambda_{i})}{\sum L_{\mathrm{source}}(\lambda_{i})}\right|)$$

This strategy extends the spectral reconstruction process from traditional static design to a dynamic power–spectrum co-optimization approach. By precisely controlling the transmittance across the full spectral range, the system maintains a stable and solar-like spectral distribution over a broad range of output power levels. Additionally, to address potential fabrication errors in high-precision multilayer designs, a transmittance tolerance band is incorporated into the filter design. This tolerance band ensures that the fabricated filter maintains compliance with spectral uniformity constraints, even in the presence of deviations in layer thickness or material refractive index, thereby enhancing the overall stability and engineering robustness of the system.

**Figure 4:**
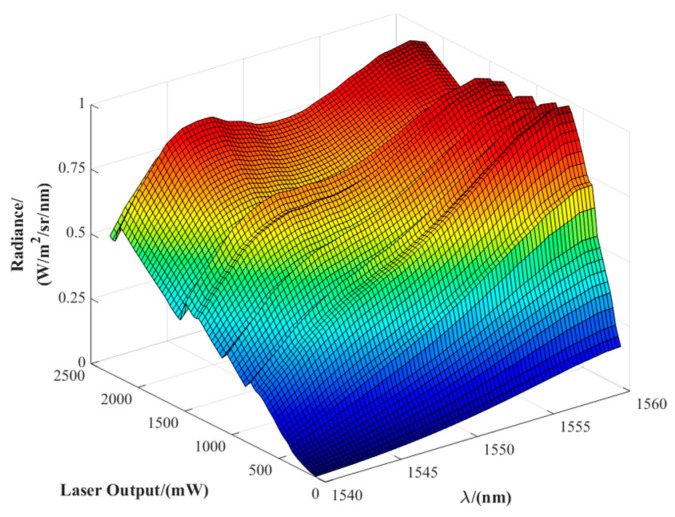
Raw spectral radiance distribution of the fiber laser across different output power levels.

**Figure 5:**
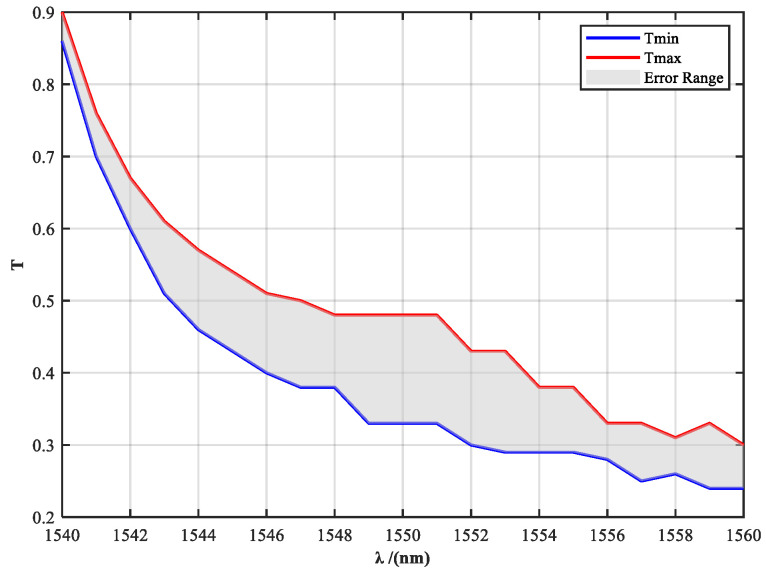
Designed spectral transmittance profile with tolerance envelope for the custom spectral shaping filter.

To define the input boundaries for filter design, the raw spectral distribution of the fiber laser source in the 1540–1560 nm band was measured and analyzed. As shown in Figure 4, the laser exhibits significant spectral drift and non-uniformity under different power levels, which fails to meet the spectral consistency requirements for accurate solar radiation simulation. According to the optimization principle described above, the transmittance tolerance curves of the spectral filter are shown in Figure 5.

### 3.3. Stray Light Suppression Analysis and Structural Design Based on BRDF Modeling

In this system, the primary source of stray light is not external environmental radiation but rather internal scattering processes induced by the solar simulation module. Since the solar background light and the signal beam are combined into a shared optical path, spatially merged via a beamsplitter and transmitted through a common optical aperture, this co-path configuration results in strong spectral and spatial overlap between internal stray radiation and the communication signal. Consequently, conventional stray light test techniques such as *VGI* and *PST* methods fail to effectively separate the two [29]. Therefore, high-precision theoretical modeling and ray-tracing simulation are required to analyze the dominant stray light paths, surface scattering mechanisms, and suppression structure performance [30]. These models form the theoretical foundation for subsequent stray energy ratio assessments and (*SNR*) optimization.

#### 3.3.1. Scattering Characterization Modeling and Energy Attenuation Mechanism Analysis

This study adopts a modeling approach based on the Bidirectional Reflectance Distribution Function (*BRDF*) to characterize the scattering properties of components [31]. For mirror-like reflective surfaces, the directional scattering characteristics are described using the ABg model, as expressed by the following Equation (9):

(9) $$BRDF=\frac{A}{B+{\left|\sin\theta_{s}-\sin\theta_{i}\right|}^{g}}$$

where $\theta_{i}$ and $\theta_{s}$ denote the incident and scattering angles, respectively; A, B, and g are model fitting parameters; Δn is the refractive index difference between incident and scattering media; and λ is the wavelength of the incident light. The total energy loss due to surface scattering is governed by the Total Integrated Scatter (*TIS*), which can be approximated as a function of the root-mean-square surface roughness *σ* using Equation (10). This model provides a quantitative basis for analyzing the impact of surface quality on system background radiation and supports surface manufacturing quality control.

(10) $$TIS={\left(\frac{2\pi \Delta n\sigma \cos\theta_{i}}{\lambda }\right)}^{2}$$

To address secondary stray light generated by non-ideal internal cavity reflections, this study introduces a path-level modeling approach and proposes a reflective light trap structure to suppress energy return. The designed U-shaped light trap is internally coated with a high-absorption material, where the single-bounce absorptance is γ. If a ray undergoes n reflections along the primary path, the residual energy ratio can be expressed as Equation (11):

(11) $$\eta ={\left(1-\gamma \right)}^{2n+1}$$

The scattering behavior of the light trap’s inner surfaces is modeled using an improved micro-surface *BRDF* formulation [32], expressed in Equation (12), where $g(\theta_{i},\varphi_{r})$ denotes the polynomial fitting function ($g_{0}$ = 0.01), which can be expressed as Formula (13), *G* is the geometric shadowing function, *F* represents the Fresnel reflection term, the exponential term describes the surface distribution function (*α* = 0.5), and the final term $k\cos\theta_{i}$ accounts for diffuse reflection.

(12) $$BRDF(\theta_{i},\varphi_{i},\theta_{r},\varphi_{r})=g(\theta_{i},\varphi_{r})\cdot \frac{G(\theta_{i},\varphi_{i},\theta_{r},\varphi_{r})}{\cos\theta_{i}\cdot \cos\theta_{r}}\cdot F\times \exp(-c^{2}\alpha^{2})+k\cos\theta_{i}$$

(13) $$g(\theta_{i},\varphi_{r})=\frac{p_{1}\cdot \varphi_{r}^{6}+p_{2}\cdot \varphi_{r}^{5}{+p}_{3}\cdot \varphi_{r}^{4}{+p}_{4}\cdot \varphi_{r}^{3}{+p}_{5}\cdot \varphi_{r}^{2}{+p}_{6}\cdot \varphi_{s}{+p}_{7}-g_{0}}{q_{1}\cdot \theta_{i}^{3}{+q}_{2}\cdot \theta_{i}^{2}{+q}_{3}\cdot \theta_{i}{+q}_{4}}{+g}_{0}$$

The theoretical modeling described above supports the subsequent experimental analysis of energy path contribution ratios, surface roughness tolerance evaluation, and structural optimization of the light trap.

Table 1 systematically defines all variables, constants, and operators used in Equations (1)–(13), along with their physical meanings and units where applicable.

#### 3.3.2. Simulation Model Construction and Parameter Configuration

To quantitatively evaluate the propagation paths and energy distribution of internal stray light, a full 3D optical model of the system was established using the ray-tracing platform TracePro. Parameterized surface scattering properties and custom ray-tracing strategies were configured accordingly.

The solar source was defined as a flat-top collimated beam at a wavelength of 1550 nm with a total power of 130 mW, corresponding to the background irradiance during Sun outage conditions. The signal light source was set as a weak, single-wavelength 1550 nm laser with a power of 1.0 × 10^−10^ W, representing an ultra-low-power communication signal. The two beams are combined within the system via a beamsplitter and propagate along a common optical path. The system output aperture was configured as the receiving surface to evaluate the power coupling ratio between background light and the signal beam. The key component parameters were set as follows: specular reflective optical surfaces were modeled using a *BRDF* scattering function, with high-reflectivity metallic coatings (reflectance: 98%). The beamsplitter was set with a transmission of 98%, reflectance of 1.8%, and absorption of 0.2%. To enable surface roughness sensitivity analysis, a scan range of 1–100 nm was defined. The inner cavity surfaces were coated with an absorptive black coating with an absorptance of 98%. The U-shaped light trap structure was modeled using an improved micro-surface *BRDF* model, and the ray tracing energy threshold was set to 10^−15^ W. During simulation, 50 million rays were traced from the solar source, with path analysis enabled to record the sequence of surfaces and types of reflections encountered by each ray. These data supported subsequent energy path clustering analysis and structural suppression optimization.

With the above modeling and simulation configuration, the system enables identification of dominant interference paths, quantitative analysis of reflection mechanisms, and performance evaluation of suppression structures. This provides theoretical support for downstream stray light energy ratio analysis and system design validation.

## 4. Experimental Validation and Results Analysis

### 4.1. Irradiance Uniformity Measurement and Evaluation

To improve the spatial energy uniformity of the solar illumination module, a composite beam shaping structure is proposed, combining a large-core homogenizing optical fiber and an apodizer. The homogenizing fiber operates based on multimode interference, effectively eliminating interference fringes and modal modulation from the laser source. The apodizer suppresses high spatial frequency components at the beam edges and reconstructs the near-field intensity distribution, enabling the formation of a quasi-flat-top irradiance profile in the far field. The apodization optics consists of three main components: a collimating lens, a spatial aperture, and a focusing lens. The key structural parameters are listed in Table 2.

To quantitatively evaluate the far-field irradiance uniformity of the homogenization structure, a measurement setup was constructed as follows. A vertical detection plane was positioned at the output aperture of the solar illumination module. A Thorlabs PM100D digital optical power meter (detector model: S145C, with an active aperture of 11.8 mm and spectral response range of 800–1700 nm) was used for energy acquisition. The power sensor was mounted on a two-dimensional precision motorized translation stage. The scanning area covered a circular region with a diameter of 200 mm, and the sampling step size was set to 10 mm in both the x and y directions to obtain the irradiance distribution over the plane.

**Figure 6:**
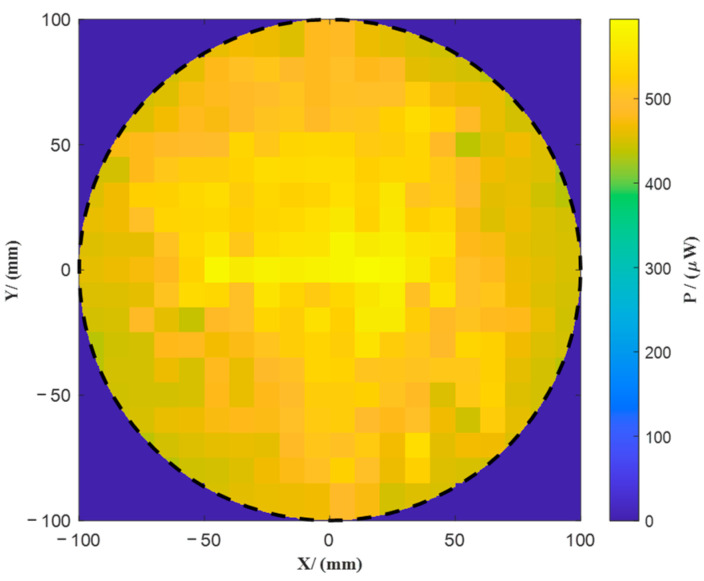
Measured far-field energy distribution.

Figure 6 shows the experimentally measured far-field energy distribution. As seen from the figure, the beam exhibits good symmetry and smoothness, forming an overall quasi-flat-top profile. Based on the irradiance uniformity evaluation Formula (14),

(14) $$U_{E}=1-\frac{E_{\max}-E_{\min}}{E_{\max}+E_{\min}}$$

where *E_max_* and *E_min_* respectively represent the maximum and minimum irradiance values within the 200 mm test area, the calculated far-field irradiance uniformity of the system reaches 85.8%. Compared to the theoretical design target of 90%, the deviation is less than 5%, indicating that the proposed homogenization optical path demonstrates excellent uniformity retention and strong engineering feasibility.

### 4.2. Measured Performance and Spectral Matching Evaluation of the Optical Filter

To verify the actual spectral modulation performance of the designed optical filter within the solar illumination module, systematic measurements were conducted using a UV–NIR spectrophotometer (Shimadzu Corporation, Kyoto, Japan) and a spectral radiometer (ASD Inc., Boulder, CO, USA). The design objective of the filter was to produce a high-uniformity, low-fluctuation spectral output within the 1540–1560 nm communication band, closely approximating the standard solar spectral distribution.

The filter transmittance curve was measured using a Shimadzu UV-3600 spectrophotometer (Shimadzu Corporation, Kyoto, Japan), with a spectral range of 185–3300 nm and a resolution of 0.5 nm. The shaped spectral distribution was measured using an ASD FieldSpec spectral radiometer (ASD Inc., Boulder, CO, USA), which covers a wavelength range of 350–2500 nm with a wavelength accuracy of ±0.5 nm. The laser output power was adjusted from 0 to 2 W in 50 mW increments, and the spectral radiance within the 1540–1560 nm band was recorded at each power level.

**Figure 7:**
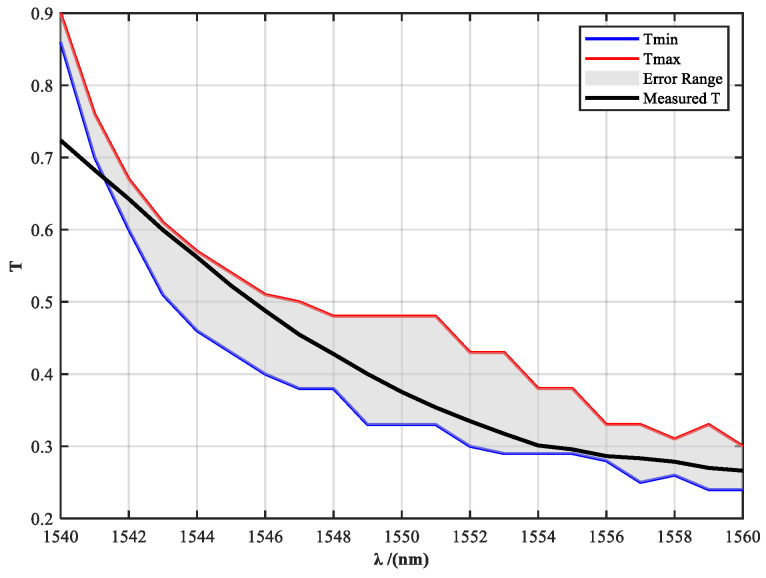
Comparison between the theoretical transmission design curve (with tolerance band) and measured results of the spectral filter.

Figure 7 presents a comparison between the theoretical design curve and the measured transmittance of the filter. The shaded area indicates the theoretical tolerance band, accounting for fabrication deviations caused by multilayer coating thickness variations. The yellow curve represents the measured transmittance of the fabricated filter sample. It can be observed that at 1540 nm and 1541 nm, the measured transmittance slightly exceeds the upper tolerance limit, resulting in deviations from the intended spectral shaping at these wavelengths. This discrepancy is mainly attributed to the high precision required in controlling the transmittance at each wavelength point, which poses significant challenges in multilayer coating fabrication and spectral matching.

**Figure 8:**
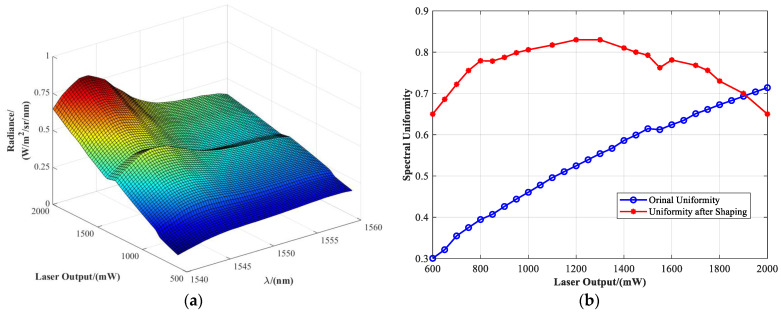
Analysis of system spectral uniformity: (**a**) curve of the spectral filter after shaping (the plot uses color gradients to represent radiance intensity); (**b**) comparison of the spectral uniformity before and after applying the filter uniformity curve.

Figure 8a shows the system’s spectral radiance distribution after spectral shaping by the designed filter, covering an output power range of 600–2000 mW. Compared to the original laser output (Figure 4), the shaped spectrum exhibits significantly improved smoothness with reduced fluctuation amplitude. The spectral consistency is notably enhanced within the typical working power range of 750–1750 mW.

To quantitatively evaluate the shaping performance, Figure 8b compares the spectral uniformity before and after applying the filter. The original laser output achieves a maximum uniformity of approximately 76% at high power (2 W), but drops below 30% at low power levels, indicating strong instability. After introducing the filter, the spectral uniformity across the 750–1750 mW range increases to over 75%, with a peak value exceeding 80% and an average spectral match of 78%. These results demonstrate that the designed filter provides effective spectral modulation and enables consistent reconstruction of the solar spectral profile across a wide power range, thereby offering a reliable foundation for realistic Sun outage background simulation.

### 4.3. Stray Light Simulation and Path Analysis

#### 4.3.1. Energy Composition Analysis of Stray Light

To evaluate the background interference level under the co-propagation structure, simulation modeling and energy ray-tracing analysis were performed separately for the solar illumination module and the signal light module. The total output energy at the system aperture can be categorized into four components: direct solar illumination, solar-induced stray light, communication signal light, and signal-induced stray light.

**Figure 9:**
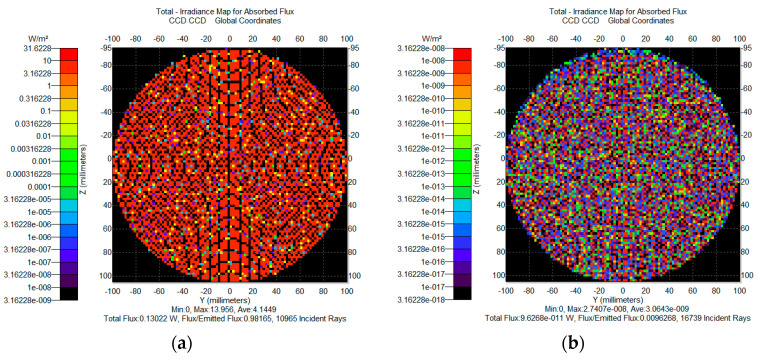
Energy composition of stray light: (**a**) solar light simulation energy; (**b**) signal light simulation energy.

For the solar illumination module, as shown in Figure 9a, the primary solar beam power is 130 mW. The main stray light caused by specular scattering paths accounts for approximately 2 × 10^−4^ W, while secondary stray light arising from multiple internal reflections within the enclosure contributes about 1 × 10^−7^ W. The signal light module, as shown in Figure 9b, delivers an effective communication signal power of approximately 9.6 × 10^−11^ W, with its own stray light component as low as 1.64 × 10^−15^ W, which is negligible relative to the signal power.

Table 3 summarizes the primary sources of stray light at the system output aperture and their corresponding energy proportions. The results indicate that the dominant source of stray light originates from specular scattering on the reflective surface of the beam splitter, accounting for 99.95% of the total stray light. The secondary transmission path, formed by solar radiation transmitted through the beam splitter into the internal cavity and subsequently reflected multiple times, contributes only 0.05% of the total stray energy. However, despite its relatively small proportion, the absolute energy level of this secondary path is significantly higher than that of the signal power and therefore cannot be neglected.

#### 4.3.2. Impact of Surface Roughness on Background Energy Levels

**Figure 10:**
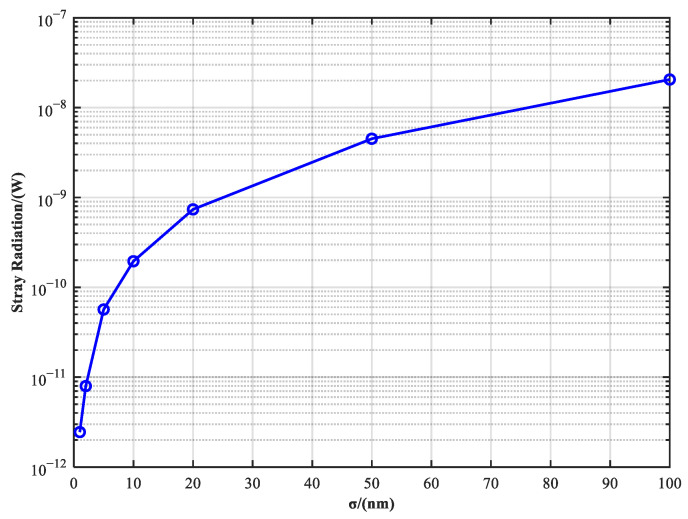
Stray radiation variation with roughness.

**Figure 11:**
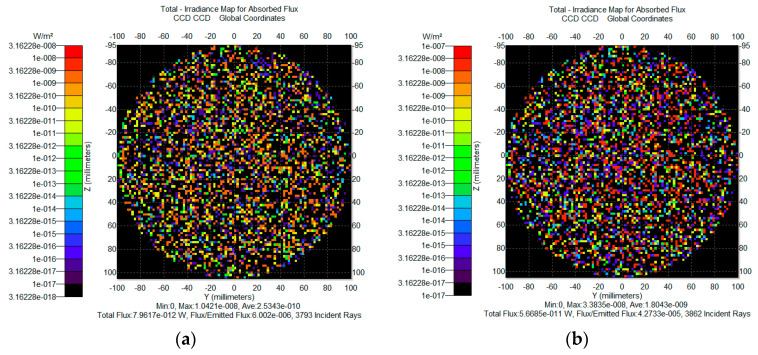
Simulation results of different roughness levels: (**a**) 2 nm specular scattering stray light; (**b**) 5 nm specular scattering stray light.

To investigate the influence of optical surface fabrication errors on the system’s SNR, a sensitivity analysis was conducted by scanning the mirror surface roughness in the range of 1–100 nm. As shown in Figure 10, the intensity of specular-scattered stray light increases significantly with surface roughness, exhibiting an exponential growth trend. When the surface roughness reaches 5 nm, the stray light power along the main path rises to 5.7 × 10^−11^ W (see Figure 11a), which is comparable to the communication signal power, resulting in the SNR dropping below 1:1 and making signal detection unfeasible. In contrast, when the roughness is controlled below 2 nm, the main-path stray light decreases to 7.96 × 10^−12^ W (see Figure 11b), achieving an SNR greater than 10:1 and meeting the system’s interference suppression requirements. These results clearly demonstrate that the background light level is highly sensitive to the surface quality of critical mirrors. Therefore, it is recommended that the surface roughness of high-reflectivity components along the primary optical path be maintained below 2 nm to ensure reliable signal recognition.

#### 4.3.3. Evaluation of the Suppression Effect of the Light Trap Structure

**Figure 12:**
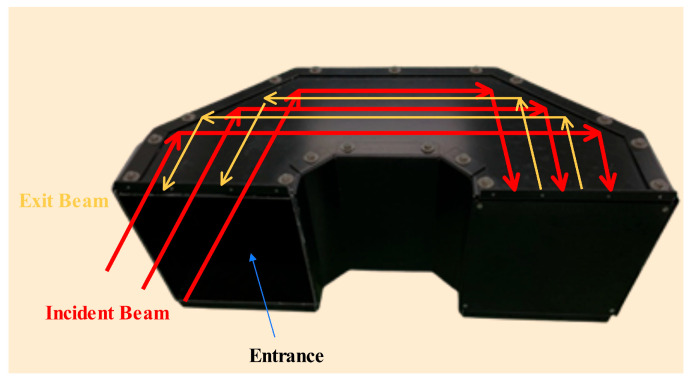
Light trap main light tracing principle and physical image.

**Figure 13:**
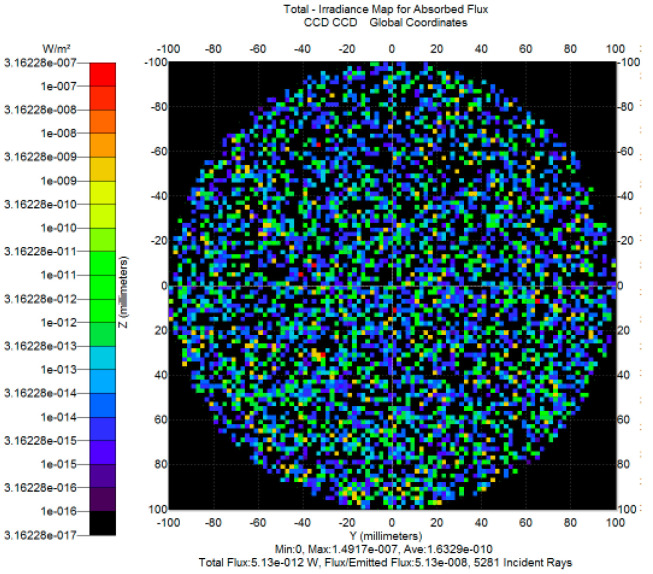
Secondary stray light simulation result.

To reduce energy leakage from secondary stray light paths caused by multiple reflections within the system cavity, a U-shaped reflective light trap was designed. The structure utilizes highly absorptive inner wall materials and an extended reflective geometry to achieve structural suppression of background illumination. The fabricated prototype and main light tracing principle, shown in Figure 12, has been assembled and demonstrates good engineering feasibility. As shown in the simulation results (Figure 13), the stray light power from secondary paths is reduced from 1.0 × 10^−7^ W to 5.13 × 10^−12^ W after introducing the light trap, achieving more than five orders of magnitude of attenuation. This confirms the effectiveness of the design in suppressing background leakage under high-interference conditions.

To ensure the accuracy of the stray light simulation model, an empirical *BRDF* model (Equation (9)) was employed to characterize the angular distribution of scattered energy from key reflective surfaces. The model parameters A, B, and g were determined through fitting with measured or typical material data, and the results are listed in Table 4. Additionally, to describe the scattering behavior of the highly absorptive inner walls of the light trap, an improved microstructure *BRDF* model (Equations (12) and (13)) was used, and the corresponding fitting parameters are presented in Table 5. The *BRDF* modeling accuracy for different materials is better than 1.5%.

### 4.4. Validation of Power Adjustment Range and Output Error

**Figure 14:**
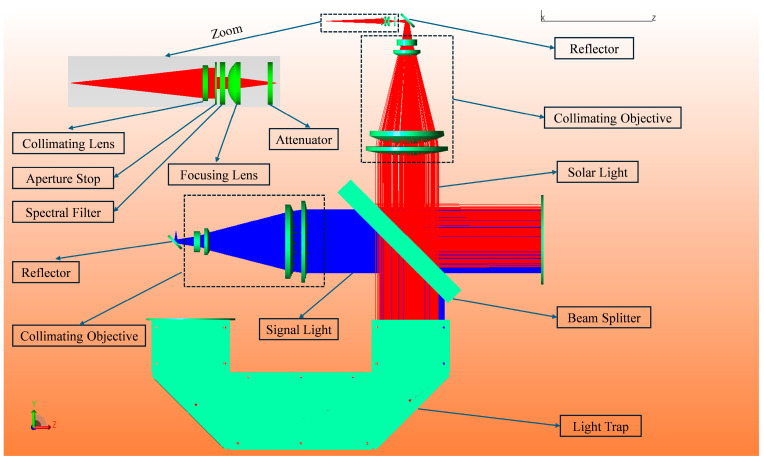
Optical design results of the solar outage simulation system.

**Figure 15:**
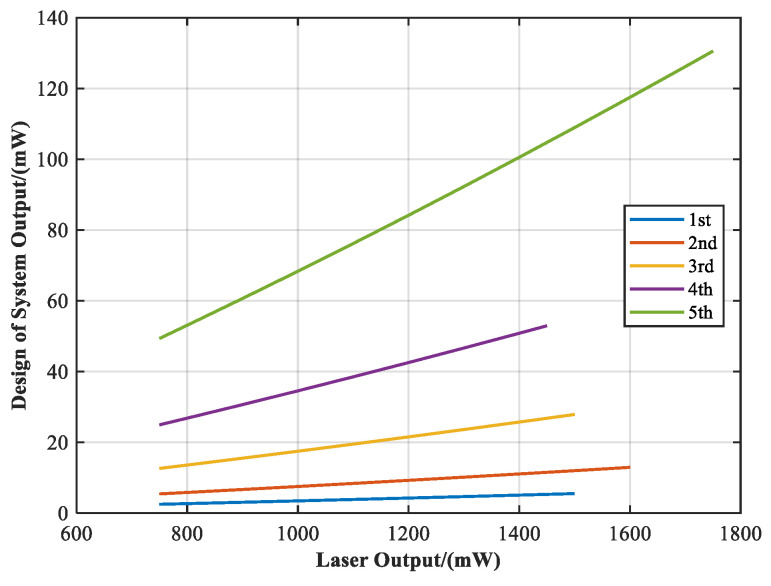
Design curve of system output variation with the change of light source output.

Although the dynamic power output range is not a unique contribution, it is indispensable for achieving high-fidelity Sun outage simulation and is therefore evaluated in detail below. To meet the requirement of dynamically controlling solar irradiance intensity for replicating Sun outage interference levels, the system incorporates a programmable attenuation module to enable multi-level continuous power output. Figure 14 presents the optical design results of the solar outage simulation system, based on the methodology detailed in preceding sections. Based on the spectral modulation by the filter and the beam homogenization structure, the transmittance of the attenuation filter set is adjusted to achieve a continuously tunable output range from 10% to 100% (max = 130 mW), as illustrated in Figure 15. Table 6 summarizes the correspondence between the transmittance of each attenuation level and the resulting system output power range.

**Figure 16:**
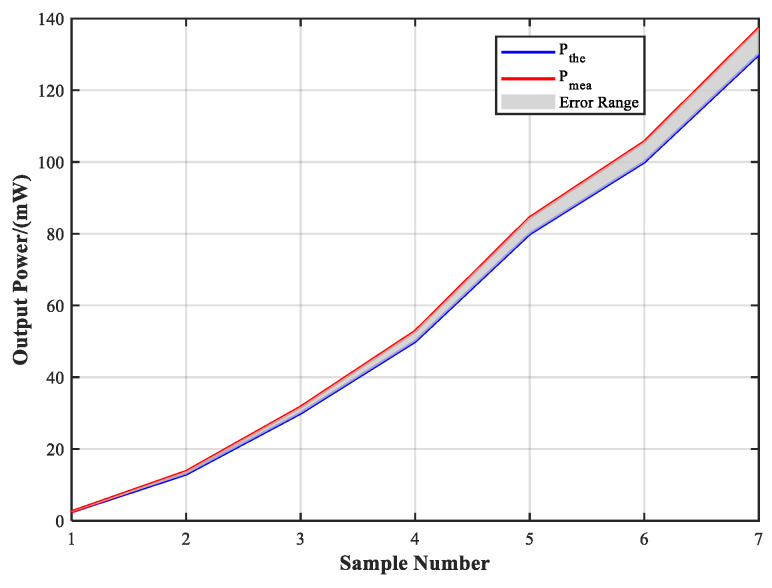
The errors curve between the theoretical and the measured power values.

To verify the power control accuracy, the system was configured with six discrete power levels spanning 2.5–130 mW. Each target output level was calibrated using an optical power meter. Figure 16 presents the deviation between the theoretical and the measured power values. The test results show that the output deviation across the full range remained within 0.9%, confirming the effectiveness of the programmable attenuation module in achieving precise power control. These results also demonstrate the system’s reliable power response capability, enabling accurate reproduction of the desired Sun outage irradiance conditions.

**Figure 17:**
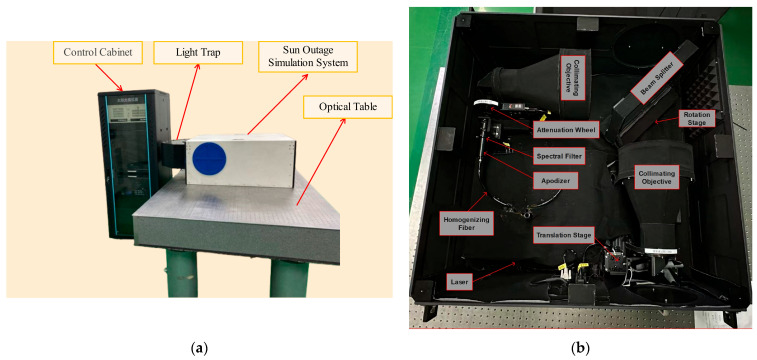
Physical diagram of Sun Outage Simulation System: (**a**) Appearance of testing system; (**b**) inside testing system.

To provide a more intuitive understanding of the engineering implementation of the proposed Sun outage simulation system, the physical setup is shown in Figure 17. The complete system consists of the solar illumination module, the signal light simulation module, and the co-propagating beam combination structure. Key components such as the homogenization unit, rotational stage, and light trap have been fully integrated and tested, ensuring stable system output performance.

## 5. Discussion

This study developed a solar radiation simulation system to meet the key requirements of ground-based Sun outage interference simulation. The system features high spectral uniformity, high irradiance homogeneity, wide-range dynamic power modulation and ultralow stray light. A co-path beam output configuration was also implemented by integrating a signal light module, enabling the simulation of realistic communication interference scenarios. Experimental validation of all key performance indicators demonstrates that the system fulfills the core parameter requirements for Sun outage simulation and exhibits both engineering feasibility and functional completeness. Nevertheless, the following limitations and areas for improvement remain:

While our *BRDF*-based ray-tracing simulations demonstrate consistent stray light suppression (5.13 × 10^−12^ W achieved in Section 4.3.3), we acknowledge the inherent limitation of relying solely on numerical modeling. The co-propagating architecture fundamentally precludes conventional stray light measurement techniques (e.g., *VGI/PST*) that require separable optical paths. Even emerging coherent detection methods, despite their high sensitivity in other applications, face three inherent constraints in our system: the 200 mm output aperture significantly exceeds the active area of available coherent detectors (<10 mm diameter) and the 10^10^ dynamic range between solar background and target stray light exceeds the linear operation range of balanced homodyne detection.

Spectral control performance is inherently constrained by filter manufacturing tolerances. Although the system achieves 78% average spectral matching (exceeding the 75% minimum requirement), localized transmittance deviations at critical edge wavelengths (1540–1541 nm) exceed theoretical predictions by ≤5%—a fundamental challenge in multilayer deposition. The power-spectrum co-optimization (Section 3.2) provides adaptive tolerance through nonlinearity compensation, maintaining >75% uniformity even with edge wavelength deviations. While the 78% mean uniformity suffices for sun outage simulation, future designs will prioritize edge wavelength control to further enhance spectral precision.

While the current system is optimized for the 1540–1560 nm communication band to address Sun outage simulation, the underlying methodology exhibits notable extensibility to broader spectral ranges. The key distinction lies in the adaptation of spectral matching criteria: our precision wavelength control approach (achieving 78% point-by-point match in narrowband) would transition to integrated irradiance matching for broadband applications (e.g., 400–2500 nm solar monitoring), where maintaining total spectral energy distribution supersedes individual wavelength accuracy. This scalability is facilitated by the modular design of both the homogenization optics (maintaining >85% uniformity regardless of spectral width) and the *BRDF*-based stray light suppression (effective across UV-NIR). However, full spectral extension would require source reconfiguration—replacing the tunable laser with a supercontinuum source and optimizing the apodizer’s chromatic performance, representing a focused area for future development.

In summary, while the system meets all its key performance objectives, further improvements are needed in spectral range scalability and stray light measurement capabilities.

## 6. Conclusions

Unlike conventional static solar simulators compliant with ASTM standards, this study develops a high-precision dynamic solar radiation platform capable of faithfully replicating transient interference dynamics during Sun outage events. By innovatively integrating a multimode-fiber-apodizer homogenization system (achieving 85.8% far-field uniformity over a 200 mm aperture), power–spectrum co-optimized filtering (78% spectral matching accuracy), and *BRDF*-modeled stray light suppression (10^−12^ W background noise), we have successfully addressed critical challenges in ground-based Sun outage simulation. The system’s 50× dynamically adjustable power range (2.5–130 mW, ±0.9% error) and inherent engineering scalability fill a key technological gap in satellite communication anti-interference testing.

## Figures and Tables

**Table 1:** Variable nomenclature for mathematical symbols.

Variables	Description	Variables	Description
E(x,y)	optical field distribution	Δn	refractive index difference between incident and scattering media
*D*	diaphragm diameter	γ	light trap absorptance
$U_{E}$	irradiation uniformity	η	light trap residual energy ratio
I(x,y)	optical intensity distribution	λ	wavelength of the incident light
*A*	cross-sectional area of the fiber core	$g(\theta_{i},\varphi_{r})$	polynomial fitting function
T(λ)	filter’s transmittance	*F*	Fresnel reflection term
$L_{\mathrm{source}}(\lambda ,P)$	the original laser spectrum output	α	surface distribution coefficient
$U_{L}$	spectral uniformity	$G(\theta_{i},\varphi_{i},\theta_{r},\varphi_{r})$	geometric shadowing function
$L_{\mathrm{solar}}(\lambda )$	the solar spectrum output	$p_{1}∼p_{7}$ $,q_{1}∼q_{4}$ $,\;g_{0}$	$\mathrm{fitting}\;\mathrm{coefficient}\;\mathrm{in}\;g(\theta_{i},\varphi_{r})$
i	index of wavelength points	k, c	fitting coefficient in micro-surface *BRDF*
j	index of power levels	ε	empirical coefficient
A, B, g	fitting coefficient in BRDF model	*TIS*	Total Integrated Scatter

**Table 2:** Parameter of apodizator (mm).

Components	Parameter of Apodizator		
	Focal Length	Effective Aperture	Mechanical Aperture
Collimating Lens	85	22	25
Aperture Stop	\ *	7	25
Focusing Lens	25	7	25

* The aperture stop serves as a mechanical limiter, with no optical focusing function, and is used solely to define the system’s input aperture.

**Table 3:** Main technical specifications of solar simulation module.

Stray Light Type	Energy Contribution (%)
Specular Scattering Stray Light	99.95
Transmissive Stray Light	0.05

**Table 4:** A, B, g model fitting parameters.

Material	Fitted Parameters			Errors (RMSE)
	A	B	g	
JGS1	0.0019	8.7679	0.5934	3.2502 × 10^−4^

**Table 5:** Improved microstructure *BRDF* model fitting parameters.

Parameters	Value	Parameters	Value	Errors (RMSE)
q_1_	0.0079	p_4_	−27.3594	0.0134
q_2_	−0.0094	p_5_	32.7085	
q_3_	0.0118	p_6_	6.6245	
q_4_	0.0241	p_7_	20.1981	
p_1_	0.0032	F	1	
p_2_	−0.0052	c	1	
p_3_	−0.2867	k	0.0048	

**Table 6:** System output with different gear (mW).

Gear	T *	Output
1	1	52.94~130.56
2	0.50	27.89~52.94
3	0.25	12.94~27.89
4	0.11	5.5~12.94
5	0.05	2.5~5.5

* T represents the transmittance of different attenuators.

## Data Availability

The data that support the findings of this study are available from the corresponding author upon reasonable request.

## Abbreviations

The following abbreviations are used in this manuscript:

OPA	Optical Parametric Amplification
LPRA	Local Pre-Rerouting Algorithm
ESA	European Space Agency
LCT	Laser Communication Terminal
NASA	National Aeronautics and Space Administration
LCRD	Laser Communications Relay Demonstration
SEP	Sun–Earth–Probe
*SNR*	Signal-to-Noise Ratio
SLM	Spatial Light Modulator
MIMI	Multimode Interference–Mode Interference
*BRDF*	Bidirectional Reflectance Distribution Function
*TIS*	Total Integrated Scatter
UV	Ultraviolet
NIR	Near Infrared
ASD	Analytical Spectral Devices
*VGI*	Veiling Glare Index
*PST*	Point Source Transmittance
ASTM	American Society for Testing and Materials
